# New 3D Vortex Microfluidic System Tested for Magnetic Core-Shell Fe_3_O_4_-SA Nanoparticle Synthesis

**DOI:** 10.3390/nano14110902

**Published:** 2024-05-21

**Authors:** Adelina-Gabriela Niculescu, Oana Maria Munteanu (Mihaiescu), Alexandra Cătălina Bîrcă, Alina Moroșan, Bogdan Purcăreanu, Bogdan Ștefan Vasile, Daniela Istrati, Dan Eduard Mihaiescu, Tony Hadibarata, Alexandru Mihai Grumezescu

**Affiliations:** 1 Department of Science and Engineering of Oxide Materials and Nanomaterials, National University of Science and Technology Politehnica Bucharest, 011061 Bucharest, Romania; adelina.niculescu@upb.ro (A.-G.N.); oanamihro@yahoo.co.uk (O.M.M.); alexandra.birca@upb.ro (A.C.B.); bogdanpb89@gmail.com (B.P.); bogdan.vasile@upb.ro (B.Ș.V.); hadibarata@curtin.edu.my (T.H.); grumezescu@yahoo.com (A.M.G.); 2 Research Institute of the University of Bucharest—ICUB, University of Bucharest, 050657 Bucharest, Romania; 3 Department of Organic Chemistry, National University of Science and Technology Politehnica Bucharest, 011061 Bucharest, Romania; alina.morosan@upb.ro (A.M.); daniela.istrati@upb.ro (D.I.); 4 BIOTEHNOS S.A., Gorunului Rue, No. 3-5, 075100 Otopeni, Romania; 5 Department of Environmental Engineering, Faculty of Engineering and Science, Curtin University Malaysia, CDT 250, Miri 98009, Malaysia

**Keywords:** microfluidic technology, 3D microreactor, vortex-type mixing, nanoparticle synthesis, core-shell magnetic nanoparticles

## Abstract

This study’s main objective was to fabricate an innovative three-dimensional microfluidic platform suitable for well-controlled chemical syntheses required for producing fine-tuned nanostructured materials. This work proposes using vortex mixing principles confined within a 3D multilayered microreactor to synthesize magnetic core-shell nanoparticles with tailored dimensions and polydispersity. The newly designed microfluidic platform allowed the simultaneous obtainment of Fe_3_O_4_ cores and their functionalization with a salicylic acid shell in a short reaction time and under a high flow rate. Synthesis optimization was also performed, employing the variation in the reagents ratio to highlight the concentration domains in which magnetite is mainly produced, the formation of nanoparticles with different diameters and low polydispersity, and the stability of colloidal dispersions in water. The obtained materials were further characterized by X-ray diffraction (XRD), Fourier-transform infrared (FT-IR) spectroscopy, dynamic light scattering (DLS), and transmission electron microscopy (TEM), with the experimental results confirming the production of salicylic acid-functionalized iron oxide (Fe_3_O_4_-SA) nanoparticles adapted for different further applications.

## 1. Introduction

Magnetic nanoparticles, which are unique and versatile materials, are broadly perceived for their important roles in various applications such as medicine, biotechnology, agriculture, engineering, and the environment [1,2,3,4,5]. These particles, usually smaller than 100 nm, obtain magnetic characteristics attributed to their tiny size and distinct compositions, including superparamagnetism and high magnetic susceptibility [5,6,7]. These nanoparticles play a crucial role in medical fields such as targeted drug delivery systems, bioimaging, biosensing, and cancer hyperthermia treatment [7,8,9,10,11]. The technology sector benefits from their utility in data storage, sensing devices, and catalysis [12,13,14,15,16].

Improving the production of magnetite nanoparticles is crucial because conventional methods, such as co-precipitation, thermal decomposition, and solvothermal processes, face challenges like uncertain size, agglomeration, and sensitivity to reaction conditions [17,18,19,20]. To overcome existing limitations, some crucial actions were proposed to improve efficiency and scalability, such as microfluidic fabrication. Using microfluidic synthesis platforms provides a controlled and reproducible environment, allows precise reaction parameter adjustment, and enhances control over particle size and morphology. Even with such emerging devices, synthesis optimization is essential for advancing toward more efficient and controlled fabrication of magnetic nanoparticles [21,22,23,24,25,26].

The most commonly encountered microfluidic configurations involve 2D geometries in which reactants meet in a horizontal plane, the same in which they are mixed, reacted, and collected. However, the channels of 2D microreactors can clog throughout Fe_3_O_4_ NP synthesis, a downfall attributed to the high reactivity of iron precursor and the large surface-to-volume ratio of the obtained materials. Other factors that contribute to particle precipitation and fouling microscale channels within conventional 2D platforms include insufficient mixing over short channel lengths and a large range of Reynolds numbers [27,28,29,30,31]. To overcome this limitation, the most convenient solution to improve mixing efficiency is to alter their geometrical patterns.

In this respect, the use of 3D microreactors in nanoparticle synthesis is a prominent development, exceeding conventional 2D microfluidic platforms by contributing to a more complex and adaptable environment. These 3D microreactors intensify control over reaction conditions and improve mixing efficiency, providing a larger surface area for a constant distribution of reactants and increasing loading capacity compared to their 2D counterparts [31,32,33]. The advanced three-dimensional geometry not only intensifies reaction kinetics for more efficient and reproducible nanoparticle synthesis but also empowers precise tuning of reaction parameters, managing superior control over particle size, morphology, and composition [34,35].

For single-phase microfluidic platforms, usual mixing principles involve spiral or zigzag channels, embedded barriers, or combinations between these variants. Such passive mixing methods are easy to employ because they avoid incorporating extra intricate components into the device. Nonetheless, in specific reactions (like those involving highly viscous fluids), sophisticated topology alone is not sufficient, requiring the inclusion of active components to boost mixing efficiency [31,36].

An alternative solution is the utilization of vortex mixing principles. The innovative vortex-type mixing mechanism in nanoparticle synthesis creates a swirling vortex within the reaction mixture, ensuring reactant mixing and homogenization [37,38]. This approach offers apparent benefits, including enhanced reaction kinetics, rapid and rigid precursor allocation, and prevention of local concentration variations for compatible nanoparticle properties [38]. Particularly advantageous for precise control over reaction parameters and the synthesis of well-defined nanoparticles, the vortex-type mixing mechanism plays a pivotal role in advancing nanoparticle synthesis methodologies. Understanding its principles and advantages provides a versatile and efficient approach adaptable to various nanoparticle fabrication processes across diverse scientific and technological applications [39,40,41].

In this context, this study aims to expand the emerging field of 3D microfluidics by proposing a novel vortex mixing type multilayer microreactor suitable for the fabrication of tunable magnetite nanoparticles. The device was designed, manufactured, and experimentally tested to produce magnetic core-shell nanoparticles (Fe_3_O_4_-SA NPs) with controlled dimensions and polydispersity. Given its appealing properties, salicylic acid (SA) was chosen for the shell layer to create a protective organic coating around the iron oxide core. Its enhanced biocompatibility, well-recognized as SA is present in natural products or as derivatives in medicines utilized by large populations [42,43], further allows the use of fabricated nanomaterials for biomedical and/or environmental applications.

To highlight the potential of the proposed device and its successful implementation for Fe_3_O_4_-SA NP synthesis, eight material variants were fabricated utilizing different Fe^3+^:Fe^2+^ molar ratios in order to optimize the Fe^3+^:Fe^2+^ ratio around the required stoichiometry and were further analyzed by a series of advanced characterization methods, including X-ray diffraction (XRD), transmission electron microscopy (TEM), dynamic light scattering (DLS), and Fourier-transform infrared spectroscopy (FT-IR).

## 2. Materials and Methods

### 2.1. Materials

For microfluidic platform fabrication, 2 mm-width polymethylmethacrylate (PMMA) sheets were used, preferably double-coated with protective foil. The working device was assembled using a commercial bicomponent epoxy adhesive (“Epoxy Universal”, Bison International B.V., Rotterdam, The Netherlands) and push-fit connectors for the required 8 mm polypropylene tubing.

The utilized materials for nanoparticle synthesis included ferric chloride (FeCl_3_) and iron sulfate heptahydrate (FeSO_4_·7H_2_O) purchased from Sigma Aldrich Merck (Darmstadt, Germany), sodium hydroxide (NaOH) purchased from Lach-Ner (Tovarni, Czech Republic), acetic acid purchased from Emsure Merck Millipore (Darmstadt, Germany), and salicylic acid purchased from ATOCHIM PROD (Bucharest, Romania). In addition, ultrapure water was utilized in all experiments. All the reagents utilized in this study were of analytical purity and used as received.

### 2.2. Microfluidic Platform Fabrication

**Figure 1:**
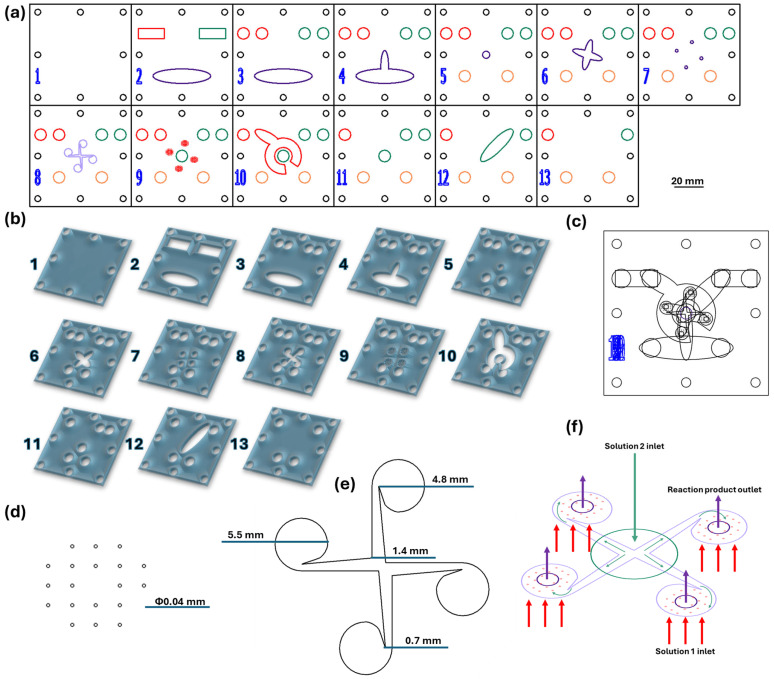
(**a**) Proportional 2D schematic representation of the microfluidic platform individual layers; Scale bar: 20 mm; (**b**) Schematic 3D representation of the microfluidic platform individual layers; (**c**) Schematic 2D overlayed view; (**d**) Reactant inlets dimensions (layer 9). (**e**) Vortex mixing chamber dimensions (layer 8); (**f**) Overlayed reaction area; red—channels for reagent solution 1 (layer 9); green—channel for reagent solution 2 (layer 9); purple—vortex mixing chamber (layer 8); violet—collecting channels (layer 7). Colors: red—inlet channels for solution 1 (iron precursors path), green—inlet channels for solution 2 (alkaline medium path), purple—vortex mixing chamber, violet—product collecting channels and chamber, orange—outlet channels.

The vortex-based mixing microfluidic chip was designed using RDWorks V8 software, which is dedicated to laser cutting machine equipment. The micromixer consists of 13 square PMMA pieces (i.e., width = length = 70 mm), with the patterns indicated in Figure 1. The model was made using the 1610 Pro laser cutting machine (RUBIQ CNC, Bacău, Romania) on 2 mm-thick PMMA sheets. The layers were overlayed, aligned, and tightened together by screws; platform edges were sealed with the epoxy adhesive, together with required inlet and outlet tubing systems.

### 2.3. Nanoparticle Synthesis

For nanoparticle fabrication, two solutions were pumped in equal volumes into the microfluidic channels using a classical osmosis pump (PSP 220 Pump, Model No. CAR6003, Water Quality Association, Lisle, IL, USA) with a 90 mL/s flow rate. Solution 1 consisted of iron oxide precursors (i.e., FeCl_3_ and FeSO_4_·7H_2_O), acetic acid, and ultrapure water, while solution 2 contained salicylic acid, NaOH, and ultrapure water, acting as both the precipitation medium and functionalization solution. In more detail, reagents were introduced through the inlets from layer 13 (marked with red for solution 1 and green for solution 2; see Figure 1a) and passed through the platform’s channels until reaching layer 8. There, iron precursors were introduced through the microfluidic channels (40 μm in diameter), while the alkaline solution was tangentially administered from the center of the layer (see Figure 1f). Furthermore, starting from layer 7, the obtained products were collected and routed toward the outlet channels (marked with orange in Figure 1a).

Different reagent ratios were employed to optimize the synthesis process; the synthesis variants are summarized in Table 1.

Reaction products were collected from the microfluidic platform outlets. Then, the obtained nanomaterials were magnetically separated by the aid of a neodymium magnet and washed with ultrapure water several times. The nanoparticles were further dispersed in ultrapure water using an ultrasonication system (750 W; 50% pulse for 10 s/break for 3 s).

### 2.4. Nanoparticle Characterization

#### 2.4.1. X-ray Diffraction (XRD)

For the XRD analysis, nanoparticle dispersions were dried, grounded as fine powders, and placed in the equipment’s sample holder. The obtained phase of the material, together with the crystallinity and crystallite dimensions, were investigated using a PANalytical Empyrean diffractometer (PANalytical, Almelo, The Netherlands) using CuKα radiation (λ = 1.5406 Å) at 40 mA and 45 kV. The diffractometer is outfitted with a hybrid monochromator (2xGe 220) located on the incident side and a parallel plate collimator mounted on a PIXcel 3D detector located on the diffracted side. Grazing incidence X-ray diffraction scans taken at room temperature covered Bragg diffraction angles of 2θ from 10° to 80° with an angle of incidence ω = 0.5°.

#### 2.4.2. Fourier-Transform Infrared Spectroscopy (FT-IR)

The obtained nanomaterials were analyzed using a Nicolet iS50FT-IR (Thermo Fisher Scientific, Waltham, MA, USA) spectrometer. The measurements were performed at ambient temperature, within the range of 4000–500 cm^−1^, using a resolution of 6 cm^−1^. All spectra were registered in the horizontal attenuated total reflectance (HATR) mode using a ZnSe crystal. In total, 96 scans were acquired for each sample, co-added, and processed using Omnic 8.2.0. (Thermo Fisher Scientific) software.

#### 2.4.3. Dynamic Light Scattering (DLS)

For the DLS investigations, the synthesized nanoparticles were dispersed in water, sonicated for 5 min, placed in dedicated cuvettes (DTS0012), and analyzed by a Nano ZS Zetasizer (Malvern Instruments, Malvern, UK). A temperature of 25 °C and a spreading angle of 90° were employed for the measurements. The values for average hydrodynamic diameter, polydispersity index, and Zeta potential have been considered as the averages of five measurements.

#### 2.4.4. Transmission Electron Microscopy (TEM) and Selected Area Electron Diffraction (SAED)

The preparation step assumed sample dispersion in ethanol by 15-min ultrasonic treatment, subsequent deposition in a small quantity onto a 400 mesh lacey carbon-coated copper grid, and air-drying at ambient temperature. High-resolution TEM micrographs were captured using a Thermo Fisher Scientific 80–200 Titan Themis transmission electron microscope (Hillsboro, OR, USA) operating at 200 kV in transmission mode, with point and line resolutions of 2 Å and 1 Å, respectively. Supplementary crystallographic data were obtained utilizing the equipment’s SAED module accessory.

## 3. Results

**Figure 2:**
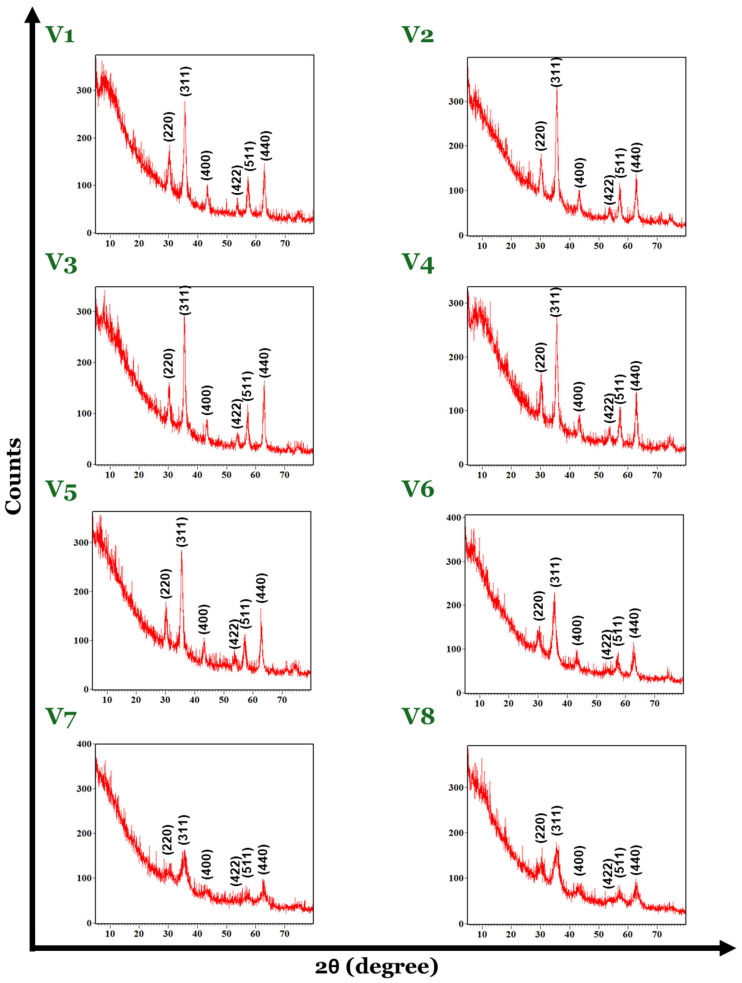
X-ray diffractograms for the eight variants of Fe_3_O_4_-SA nanoparticles.

The XRD pattern of the magnetite nanoparticles shows the presence of peaks at the 2θ values of 30.18°, 35.42°, 43.22°, 53.62°, 57.18°, and 62.85°, corresponding to the (220), (311), (400), (422), (511), and (440) planes, which are characteristic for the magnetite as a single phase (Figure 2). According to the PDF-ICDD database, the cubic spinel crystallographic system was identified for the magnetite samples from V1 to V7 with the Fd-3 m space group, whereas for the V8 magnetite, it corresponds to the orthorhombic crystallographic system with the Pmc21 space group. The peaks observed at the 2θ values decreased when using a Fe^3+^:Fe^2+^ molar ratio greater than 2:1.2 (V6–V8), while, by using a Fe^3+^:Fe^2+^ molar ratio between approximately 1:2 and 2:1, the material keeps the specific crystalline structure of spinel (V1–V5). The characteristics of the peaks in the case of samples V6–V8 in terms of their width are greater compared to samples V1–V5, which may indicate a smaller crystallite size. However, the crystalline feature of the magnetite samples obtained by varying synthesis parameters is observed for all eight XRD data results.

For the V1–V8 magnetite crystallite size evaluation in terms of average size expressed in nanometers, the Debye–Scherrer formula was applied, as follows:

$$D=\frac{0.9\lambda }{\beta cos\theta }$$

In this mathematical calculation, the crystallite size “*D*” is obtained by reporting the values of the “λ”, represented by the wavelength (0.1540 nm) of the X-ray specified for the equipment, “β”, which is related to the full width at half maximum (FWHM), and “θ” as the diffraction angle.

The results are included in Table 2, representing the average crystallite size expressed in nanometers for each sample of the V1–V8 magnetite set.

The maximum value for crystallite size was obtained for the V3 sample, 15.08 nm, while the minimum value was 4.55 nm for the V8 magnetite sample. The X-ray diffraction patterns indicated the presence of a smaller crystallite size, which was confirmed by the results obtained through the Debye–Scherrer formula.

**Figure 3:**
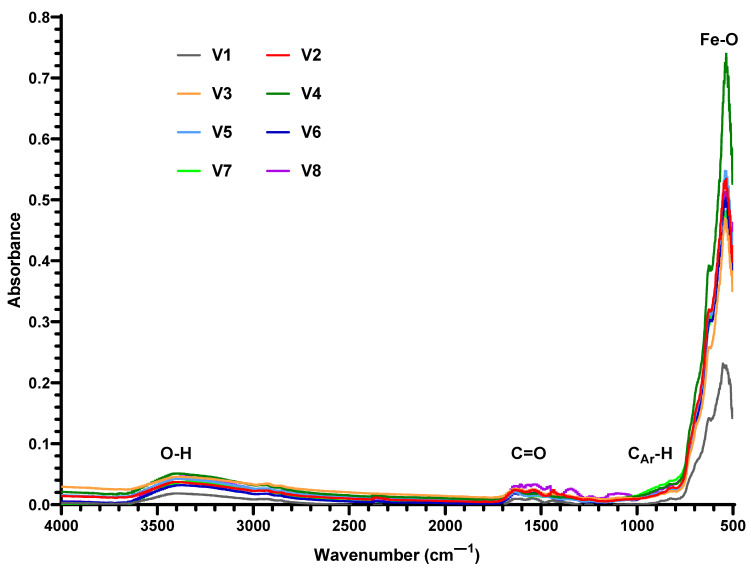
FT-IR spectra for the eight variants of Fe_3_O_4_-SA nanoparticles.

The success of the functionalization approach was verified by FT-IR spectroscopy for all the synthesized materials (Figure 3). The existence of the SA shell was confirmed in the FT-IR spectrum by the presence of the characteristic band for the -OH stretching vibrations at 3344–3416 cm^−1^, the C=O stretching band of the carboxyl group at 1603–1636 cm^−1^, and the characteristic absorption band for asymmetric stretching vibrations of carboxylate groups at 1535–1557 cm^−1^. The aromatic structure of SA is evidenced by characteristic bands ranging from 817–829 and 870–879 cm^−1^. The O-H bending band from the carboxylic group can be observed at 1437–1473 cm^−1^ and the characteristic band assigned to the Fe-O bond vibration at 535 cm^−1^.

Advanced characterization techniques such as dynamic light scattering (DLS) were used to evaluate the hydrodynamic diameter, the zeta potential, and the polydispersity of the V1–V8 set of Fe_3_O_4_-SA samples.

**Figure 4:**
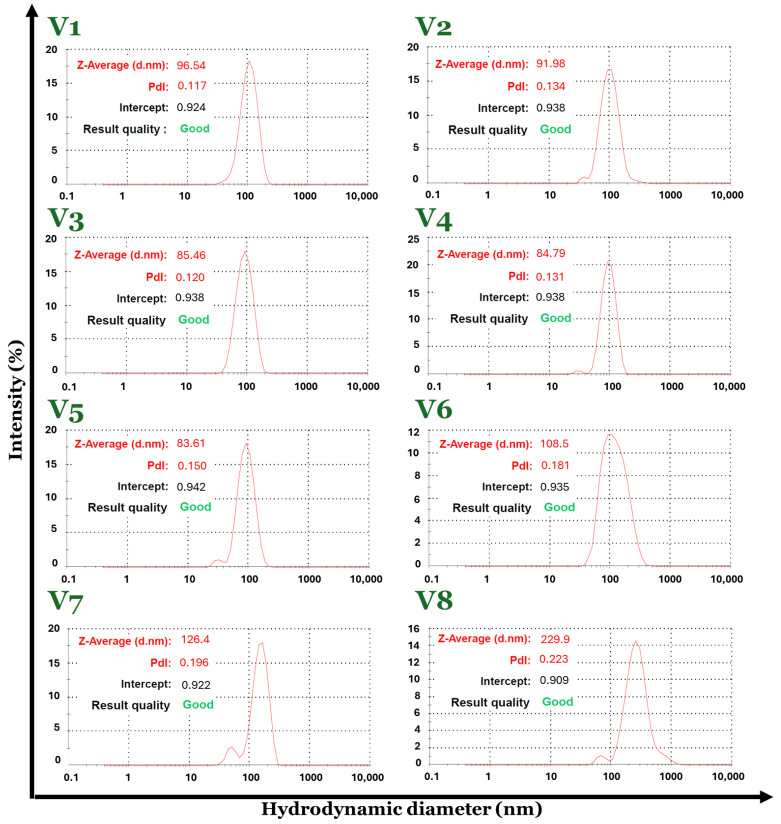
Size distribution by intensity for the eight variants of Fe_3_O_4_-SA nanoparticles.

**Figure 5:**
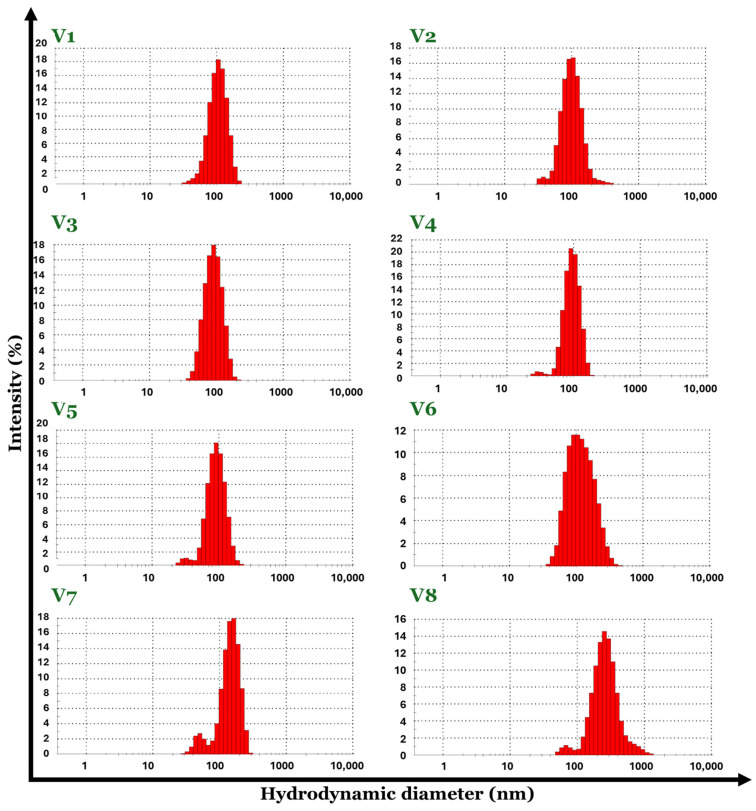
Statistics graph for the eight variants of Fe_3_O_4_-SA nanoparticles.

**Figure 6:**
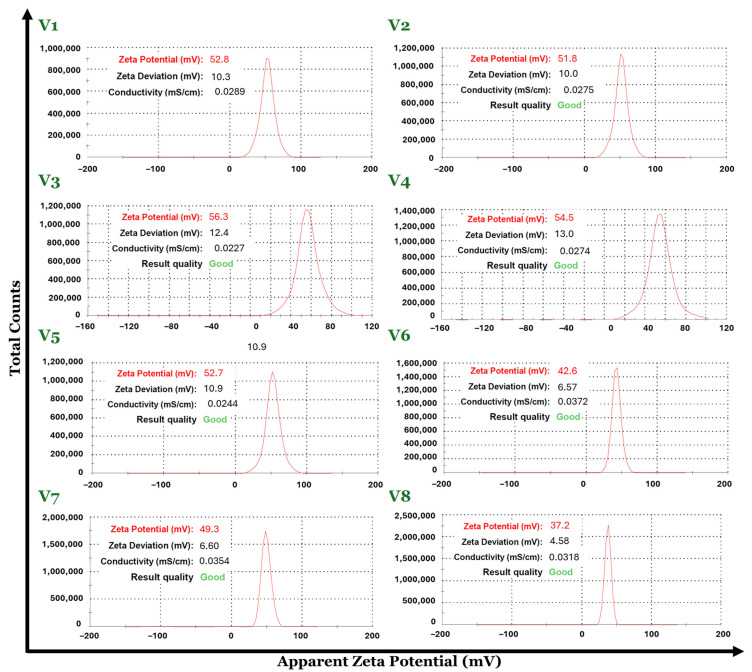
Zeta potential distribution for the eight variants of Fe_3_O_4_-SA nanoparticles.

The Fe_3_O_4_-SA (V1–V8) nanoparticle suspensions in water possess a hydrodynamic diameter with values in the range of 83–229 nm. By comparing the DLS results in terms of hydrodynamic diameter, the smallest value was recorded for the V5 sample (83.61 nm) and the biggest value is attributed to the V8 sample (229.9 nm). Considering the fact that the hydrodynamic diameter is about 10 times higher than the physical diameter of the particles, it may be concluded that V8 has increased the size dimensions of the particles regarding the synthesis parameters, which directly influence the physicochemical properties of the material. Moreover, the Fe_3_O_4_-SA (V1–V8) nanoparticles exhibit good size homogeneity with a narrow size distribution due to their very low polydispersity with values between 0.117 and 0.233 Mw/Mn (Figure 4 and Figure 5). The zeta potential values were positive for all eight formulations of Fe_3_O_4_-SA, indicating that nanoparticles possess a positive charge on their surface. In terms of colloidal stability, the resulting zeta potential data show considerable properties with values between 37 (V8) and 56 (V3) mV without the sedimentation process of the particles (Figure 6).

All data obtained from DLS analysis indicate the influence of the synthesis parameters used in obtaining the Fe_3_O_4_-SA (V1–V8) nanoparticles, highlighting the fact that by using Fe^3+^:Fe^2+^ molar ratios between approximately 1:2 and 2:1, the size of the nanoparticles does not vary significantly and the polydispersity index remains low. Also, the stability of the dispersions is very good according to the zeta potential of over 50 mV. Exceeding the 2:1 Fe^3+^:Fe^2+^ molar ratio leads to nanoparticles with hydrodynamic diameters greater than 100 nm and lower zeta potential values.

**Figure 7:**
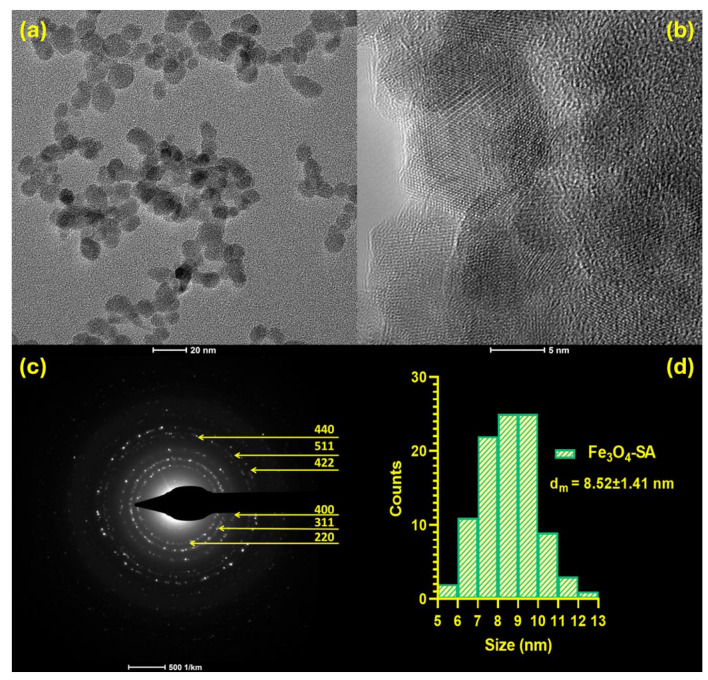
(**a**,**b**) TEM micrographs, (**c**) SAED pattern, and (**d**) size distribution of Fe_3_O_4_-SA (V3) nanoparticles.

The morphology of Fe_3_O_4_-SA NPs (V3) was determined by transmission electron microscopy (Figure 7). The bright-field and high-resolution TEM micrographs (Figure 7a,b) confirm the presence of uniform nanoparticles with exclusive spherical morphology and reduced aggregation tendency. Based on the size distribution of nanoparticles (Figure 7d), the average particle size of synthesized Fe_3_O_4_-SA was determined at 8.52 ± 1.41 nm, with the nanomaterials presenting a monomodal size distribution. The crystallite size value for the V3 sample was 15.08 nm, suggesting that the material is monocrystalline and organized as one crystallite. Moreover, the hydrodynamic diameter for the V3 magnetite sample was 85.46 nm, which is correlated with the physical dimension of 8.52 nm. In addition, the selected area electron diffraction (SAED) pattern (Figure 7c) proves the crystalline structure of the developed nanomaterial, with the concentric rings formed at (220), (311), (400), (422), (511), and (440), matching the planes determined by XRD analysis, thus confirming the magnetite as single phase.

## 4. Discussion

Compared with our prior work on 3D microfluidic platforms [44], this study has demonstrated the simultaneous synthesis and functionalization of magnetite nanoparticles with salicylic acid, proposing a fast and efficient procedure for fabricating core-shell nanostructures that eliminates lengthy intermediate processing stages. The proposed multilayered configuration with reactants in vortex flow in four chambers with multichannel feeding and subsequent continuous mixing in a secondary post-reaction processing chamber enabled the production of uniform Fe_3_O_4_-SA nanoparticles in a short time. The use of 20 inlet channels with a small diameter (40 μm) for each reaction chamber compensated for potential problems related to the high-pressure drop. Another significant advantage is related to the system’s operation under high flow rates, which yields high productivity compared to other synthetic alternatives. The rapid mixing of the reactants in a high turbulence regime allowed good contact between the iron precursors and the alkaline solution containing the functionalization agent, leading to a high throughput of homogenous core-shell magnetic nanoparticles, also involving the auto-assembling of the organic shell of the NPs.

This study aimed to optimize the synthesis of magnetic nanoparticles with a focus on improving efficiency and scalability, ultimately advancing applications in medicine, engineering, food, and agriculture. Given that the behavior of Fe_3_O_4_ NPs is closely related to their size, morphology, and surface chemistry, the production procedure demands simplicity, reproducibility, and repeatability toward obtaining uniform nanoparticles with tailored properties [18,45,46,47]. The use of microfluidic platforms has been considered in this context, with several devices being reported for the on-chip fabrication of Fe_3_O_4_ NPs [18]. Nonetheless, existent microreactors have predominantly tackled two-dimensional mixing approaches, with microfluidic 3D mixing still being in its infancy [31]. To our knowledge, the only 3D microfluidic platform successfully tested for Fe_3_O_4_ NP production is represented by our previously elaborated device [44], in which bare iron oxide nanoparticles were synthesized on-chip and were further functionalized by microwave-assisted silanization.

As demonstrated by DLS and TEM analyses, the obtained Fe_3_O_4_-SA NPs were uniform, with narrow size distribution and spherical morphology. This shape is commonly observed in microfluidic synthesis methods, with many studies documenting the production of Fe_3_O_4_-based nanospheres on-chip [27,48,49,50,51]. However, by carefully adjusting the operational parameters (such as reagent flow, residence time, temperature, and channel geometry), it is possible to obtain different morphologies, including octahedral-shaped nanocrystals [52], hexagonal plates [52], and tadpole-like particles [53].

Magnetic nanoparticles are known to agglomerate and oxidize quickly, processes that may alter their desirable characteristics and impede their further applications [54,55]. Thus, their surface chemistry is usually altered with the aid of various natural and synthetic compounds (e.g., polymers, metals, or organic and/or inorganic stabilizing agents) [18,54]. From the wide range of possible functionalization agents, we have chosen salicylic acid for this work based on our previous experience [56,57,58,59]. The successful functionalization of nanoparticles was confirmed through FT-IR analyses, with the SA shell being present on all eight nanocomposite variants. One significant benefit of SA is the potential ionization of the phenolic OH group following the shell formation stage. By utilizing the ionization step, the polarity of the magnetic nanoparticle shell can be adjusted from weak polar to ionic, allowing for water dispersibility and long-term stability. The stability of the nanomaterial is also ensured by the remarkable interaction between the carboxylic anion from SA and the iron oxide core [57].

In addition to building the SA shell during the Fe_3_O_4_ synthesis, this study has also investigated the impact of the initial molar ratio of Fe^3+^:Fe^2+^ on the phase composition, crystallinity, size, and polydispersity. XRD analyses revealed that by using a Fe^3+^:Fe^2+^ molar ratio between approximately 1:2 and 2:1, the obtained materials (V1–V5) are crystalline with a spinel structure characteristic of magnetite while at a molar ratio greater than 2:1.2 (V6–V8), the peaks are less pronounced. Moreover, based on DLS results, between 1:2 and 2:1 Fe^3+^:Fe^2+^ molar ratio variants (V1–V5), there is no significant variance in size and polydispersity index and the zeta potential values are over 50mV. After exceeding the 2:1 Fe^3+^:Fe^2+^ molar ratio, the hydrodynamic diameters rise above 100 nm and the zeta potentials decrease.

Magnetite demonstrates superparamagnetism as its size decreases within the single-domain range of around 20 nm. This characteristic is crucial for its utilization in biomedical applications [60,61,62]. With an average particle size of 8.52 ± 1.41 nm (determined by TEM analysis), the synthesized Fe_3_O_4_-SA NPs fit within the desirable dimensional range, suggesting the applicability of the obtained materials for bionanomedicine purposes. Moreover, studies have indicated the potential of Fe_3_O_4_-SA NPs of comparable hydrodynamic diameters with those obtained by our DLS analysis (V1–V5) for several biomedical uses, including the management of bleeding-induced anemia [63], melanoma [64,65], and vascular nanoblockages [66].

Surface-functionalized Fe_3_O_4_ NPs have also been recognized as valuable materials for water decontamination purposes, acting as effective nanoadsorbents for various pollutants and enabling facile magnetic separation from aqueous samples after treatment [2]. Specifically, prior studies reported the successful utilization of citric acid-coated Fe_3_O_4_ NPs for crystal violet adsorption [54], aminochitosan-coated Fe_3_O_4_ NPs for diclofenac sodium adsorption [67], polyaniline-coated Fe_3_O_4_ NPs with for the adsorption of polycyclic aromatic hydrocarbons [68], silica-coated Fe_3_O_4_ NPs for Pb(II) adsorption [69], and [68] salicylic acid-coated Fe_3_O_4_ NPs for Cd(II) adsorption [70]. Therefore, future research studies may be oriented toward testing the developed nanocomposites for removing various compounds from contaminated water samples, thus checking their utility and efficacy for environmental remediation applications.

## 5. Conclusions

This study proposed an innovative device for generating core-shell nanoparticles utilizing the emerging technology of 3D microfluidic platforms and vortex mixing principles, offering a more efficient alternative to conventional synthesis methods and typical microchips. The flexibility and capability of the 3D vortex-type multilayered microreactor platform for fast micromixing have been demonstrated through the production of uniform Fe_3_O_4_-SA nanoparticles in a short time. The experimental results highlight the concentration domains in which magnetite is mainly produced, the formation of nanoparticles with different diameters and low polydispersity, and high water colloidal dispersibility. All proposed nanoparticle variants were successfully functionalized with salicylic acid and FT-IR analyses confirmed the existence of the shell. Based on XRD and DLS analyses, desirable crystallinity, polydispersity, hydrodynamic diameters, and stability of fabricated nanoparticles are obtained for Fe^3+^:Fe^2+^ molar ratios between approximately 1:2 and 2:1 (V1–V5). In addition, TEM investigations revealed the formation of Fe_3_O_4_-SA nanoparticles of homogeneous size and morphology, with the obtained nanospheres having an average diameter of 8.52 ± 1.41 nm. Thus, the experimental results confirmed the production of magnetic nanoparticles adapted for different further applications, especially in the fields of biomedicine and environmental remediation.

## Figures and Tables

**Table 1:** Modifications employed in reagent molar ratios for synthesis optimization.

**Variant**	V1	V2	V3	V4	V5	V6	V7	V8
**Molar ratio (Fe^3+^:Fe^2+^)**	1:1.95	1:1.30	2:1.66	2:1.46	2:1.16	3:1.46	3:1.17	4:1.17

**Table 2:** Crystallite size comparison between the eight variants of Fe_3_O_4_-SA nanomaterials.

**Sample**	V1	V2	V3	V4	V5	V6	V7	V8
**Average crystallite size (nm)**	14.70	10.13	15.08	14.76	9.53	8.84	4.75	4.55

## Data Availability

The data presented in this study are available on request from the authors.

## References

[1] Kudr J., Haddad Y., Richtera L., Heger Z., Cernak M., Adam V., Zitka O. (2017). Magnetic Nanoparticles: From Design and Synthesis to Real World Applications. Nanomaterials.

[2] Niculescu A.-G., Mihaiescu B., Mihaiescu D.E., Hadibarata T., Grumezescu A.M. (2024). An Updated Overview of Magnetic Composites for Water Decontamination. Polymers.

[3] Rani K., Singh J., Jangra A., Kumar J., Kumar P., Kumar S., Singh D., Kumar R. (2023). Kinetics and isotherm investigations on the improved adsorption of the antibiotic moxifloxacin from aqueous solution utilizing agar coated magnetite nanoparticles. Biointerface Res. Appl. Chem..

[4] Mostafapour F.K., Miri A., Khatibi A., Balarak D., Kyzas G.Z. (2023). Survey of Fe_3_O_4_ Magnetic Nanoparticles Modified with Sodium Dodecyl Sulfate for Removal P-Cresol and Pyrocatechol from Aqueous Solutions. Biointerface Res. Appl. Chem..

[5] Nguyen M.D., Tran H.-V., Xu S., Lee T.R. (2021). Fe_3_O_4_ Nanoparticles: Structures, Synthesis, Magnetic Properties, Surface Functionalization, and Emerging Applications. Appl. Sci..

[6] Akbarzadeh A., Samiei M., Davaran S. (2012). Magnetic nanoparticles: Preparation, physical properties, and applications in biomedicine. Nanoscale Res. Lett..

[7] Asefi Y., Fahimi R., Ghorbian S. (2021). Synergistic effect of vitamin c with superparamagnetic iron oxide nanoparticles for inhibiting proliferation of gastric cancer cells. Biointerfaces Res. Appl. Chem..

[8] Włodarczyk A., Gorgoń S., Radoń A., Bajdak-Rusinek K. (2022). Magnetite Nanoparticles in Magnetic Hyperthermia and Cancer Therapies: Challenges and Perspectives. Nanomaterials.

[9] Baranwal J., Barse B., Di Petrillo A., Gatto G., Pilia L., Kumar A. (2023). Nanoparticles in Cancer Diagnosis and Treatment. Materials.

[10] Materón E.M., Miyazaki C.M., Carr O., Joshi N., Picciani P.H.S., Dalmaschio C.J., Davis F., Shimizu F.M. (2021). Magnetic nanoparticles in biomedical applications: A review. Appl. Surf. Sci. Adv..

[11] Jalalvand M., Falahzadeh K., Jalalvand A., Mazloumi M., Shahsavari G. (2023). Optimization of the Expression of Recombinant Cetuximab Single-Chain Fragment Variable and Comparative its Purification with Magnetic Nanoparticles and Conventional Fast Protein Liquid Chromatography. Biointerface Res. Appl. Chem..

[12] Picchi D.F., Biglione C., Horcajada P. (2023). Nanocomposites Based on Magnetic Nanoparticles and Metal–Organic Frameworks for Therapy, Diagnosis, and Theragnostics. ACS Nanosci. Au.

[13] Liao Z., Zoumhani O., Boutry C.M. (2023). Recent Advances in Magnetic Polymer Composites for BioMEMS: A Review. Materials.

[14] Gerasimov E. (2022). Synthesis of Nanocomposites and Catalysis Applications. Nanomaterials.

[15] Kar D.K., Praveenkumar V., Si S., Panigrahi H., Mishra S. (2024). Carbon Dots and Their Polymeric Nanocomposites: Insight into Their Synthesis, Photoluminescence Mechanisms, and Recent Trends in Sensing Applications. ACS Omega.

[16] Gerasimov E. (2023). Synthesis of Nanocomposites and Catalysis Applications II. Nanomaterials.

[17] Majidi S., Zeinali Sehrig F., Farkhani S.M., Soleymani Goloujeh M., Akbarzadeh A. (2016). Current methods for synthesis of magnetic nanoparticles. Artif. Cells Nanomed. Biotechnol..

[18] Niculescu A.-G., Chircov C., Grumezescu A.M. (2022). Magnetite nanoparticles: Synthesis methods—A comparative review. Methods.

[19] Díez A.G., Rincón-Iglesias M., Lanceros-Méndez S., Reguera J., Lizundia E. (2022). Multicomponent magnetic nanoparticle engineering: The role of structure-property relationship in advanced applications. Mater. Today Chem..

[20] Mittal N., Kundu A., Pathania A.R. (2023). A review of the chemical synthesis of magnetic nano-particles and biomedical applications. Mater. Today Proc..

[21] Schemberg J., Abbassi A.E., Lindenbauer A., Chen L.-Y., Grodrian A., Nakos X., Apte G., Khan N., Kraupner A., Nguyen T.-H. (2022). Synthesis of Biocompatible Superparamagnetic Iron Oxide Nanoparticles (SPION) under Different Microfluidic Regimes. ACS Appl. Mater. Interfaces.

[22] Chircov C., Dumitru I.A., Vasile B.S., Oprea O.-C., Holban A.M., Popescu R.C. (2023). Microfluidic Synthesis of Magnetite Nanoparticles for the Controlled Release of Antibiotics. Pharmaceutics.

[23] Bezelya A., Küçüktürkmen B., Bozkır A. (2023). Microfluidic Devices for Precision Nanoparticle Production. Micro.

[24] Kulkarni M.B., Goel S. (2020). Microfluidic devices for synthesizing nanomaterials—A review. Nano Express.

[25] Li Z., Zhang B., Dang D., Yang X., Yang W., Liang W. (2022). A review of microfluidic-based mixing methods. Sens. Actuators A Phys..

[26] Mohammadi M., Ahmed Qadir S., Mahmood Faraj A., Hamid Shareef O., Mahmoodi H., Mahmoudi F., Moradi S. (2024). Navigating the future: Microfluidics charting new routes in drug delivery. Int. J. Pharm..

[27] Bemetz J., Wegemann A., Saatchi K., Haase A., Häfeli U.O., Niessner R., Gleich B., Seidel M. (2018). Microfluidic-Based Synthesis of Magnetic Nanoparticles Coupled with Miniaturized NMR for Online Relaxation Studies. Anal. Chem..

[28] James M., Revia R.A., Stephen Z., Zhang M. (2020). Microfluidic Synthesis of Iron Oxide Nanoparticles. Nanomaterials.

[29] Vasilescu S.A., Bazaz S.R., Jin D., Shimoni O., Warkiani M.E. (2020). 3D printing enables the rapid prototyping of modular microfluidic devices for particle conjugation. Appl. Mater. Today.

[30] Wang J., Zhang N., Chen J., Rodgers V.G.J., Brisk P., Grover W.H. (2019). Finding the optimal design of a passive microfluidic mixer. Lab Chip.

[31] Niculescu A.-G., Mihaiescu D.E., Grumezescu A.M. (2022). A Review of Microfluidic Experimental Designs for Nanoparticle Synthesis. Int. J. Mol. Sci..

[32] Tan Z., Shi H., Zheng Y., Cao Y. (2023). A 3D homogeneous microreactor with high mixing intensity at wide Re range for MOF preparation and POCT application. Chem. Eng. J..

[33] Wang Y., Seidel M. (2021). Integration of 3D Hydrodynamic Focused Microreactor with Microfluidic Chemiluminescence Sensing for Online Synthesis and Catalytical Characterization of Gold Nanoparticles. Sensors.

[34] Aguirre-Cortés J.M., Moral-Rodríguez A.I., Bailón-García E., Davó-Quiñonero A., Pérez-Cadenas A.F., Carrasco-Marín F. (2023). 3D printing in photocatalysis: Methods and capabilities for the improved performance. Appl. Mater. Today.

[35] Middelkoop V., Slater T., Florea M., Neațu F., Danaci S., Onyenkeadi V., Boonen K., Saha B., Baragau I.-A., Kellici S. (2019). Next frontiers in cleaner synthesis: 3D printed graphene-supported CeZrLa mixed-oxide nanocatalyst for CO_2_ utilisation and direct propylene carbonate production. J. Clean. Prod..

[36] Koryakina I.G., Afonicheva P.K., Arabuli K.V., Evstrapov A.A., Timin A.S., Zyuzin M.V. (2021). Microfluidic synthesis of optically responsive materials for nano- and biophotonics. Adv. Colloid Interface Sci..

[37] Hakke V., Sonawane S., Anandan S., Sonawane S., Ashokkumar M. (2021). Process Intensification Approach Using Microreactors for Synthesizing Nanomaterials—A Critical Review. Nanomaterials.

[38] Liu Y., Cheng C., Liu Y., Prud’homme R.K., Fox R.O. (2008). Mixing in a multi-inlet vortex mixer (MIVM) for flash nano-precipitation. Chem. Eng. Sci..

[39] Liu L., Yang X., Guo Y., Li B., Wang L.-P. (2023). Reactive mixing performance for a nanoparticle precipitation in a swirling vortex flow reactor. Ultrason. Sonochem..

[40] Khan I., Saeed K., Khan I. (2019). Nanoparticles: Properties, applications and toxicities. Arab. J. Chem..

[41] Kumari S., Raturi S., Kulshrestha S., Chauhan K., Dhingra S., András K., Thu K., Khargotra R., Singh T. (2023). A comprehensive review on various techniques used for synthesizing nanoparticles. J. Mater. Res. Technol..

[42] Mishra A.K., Baek K.-H. (2021). Salicylic Acid Biosynthesis and Metabolism: A Divergent Pathway for Plants and Bacteria. Biomolecules.

[43] Mikhnavets L., Abashkin V., Khamitsevich H., Shcharbin D., Burko A., Krekoten N., Radziuk D. (2022). Ultrasonic Formation of Fe_3_O_4_-Reduced Graphene Oxide–Salicylic Acid Nanoparticles with Switchable Antioxidant Function. ACS Biomater. Sci. Eng..

[44] Niculescu A.-G., Moroșan A., Bîrcă A.C., Gherasim O., Oprea O.C., Vasile B.Ș., Purcăreanu B., Mihaiescu D.E., Rădulescu M., Grumezescu A.M. (2023). Microwave-Assisted Silanization of Magnetite Nanoparticles Pre-Synthesized by a 3D Microfluidic Platform. Nanomaterials.

[45] Fatmawati T., Shiddiq M., Armynah B., Tahir D. (2023). Synthesis Methods of Fe_3_O_4_ Nanoparticles for Biomedical Applications. Chem. Eng. Technol..

[46] Venkatesh N., Kumar N.H., Goud S., Ravinder D., Somaiah P.V., Babu T.A., Prasad N.V.K. (2021). FTIR, optical, electrical and magnetic properties of SM3+ doped MG nano ferrites. Biointerface Res. Appl. Chem..

[47] Selima S.S., Bayoumy W.A., Khairy M., Mousa M.A. (2021). Structural, Magnetic, Optical Properties and Photocatalytic Activity of Nanocrystalline Cobalt Ferrite Prepared by Three Different Methods. Res. Sq..

[48] Kašpar O., Koyuncu A.H., Hubatová-Vacková A., Balouch M., Tokárová V. (2020). Influence of channel height on mixing efficiency and synthesis of iron oxide nanoparticles using droplet-based microfluidics. RSC Adv..

[49] Kumar K., Nightingale A.M., Krishnadasan S.H., Kamaly N., Wylenzinska-Arridge M., Zeissler K., Branford W.R., Ware E., deMello A.J., deMello J.C. (2012). Direct synthesis of dextran-coated superparamagnetic iron oxide nanoparticles in a capillary-based droplet reactor. J. Mater. Chem..

[50] Ohannesian N., De Leo C.T., Martirosyan K.S. (2019). Dextran coated superparamagnetic iron oxide nanoparticles produced by microfluidic process. Mater. Today Proc..

[51] Chircov C., Bîrcă A.C., Grumezescu A.M., Vasile B.S., Oprea O., Nicoară A.I., Yang C.-H., Huang K.-S., Andronescu E. (2021). Synthesis of Magnetite Nanoparticles through a Lab-On-Chip Device. Materials.

[52] Larrea A., Sebastian V., Ibarra A., Arruebo M., Santamaria J. (2015). Gas slug microfluidics: A unique tool for ultrafast, highly controlled growth of iron oxide nanostructures. Chem. Mater..

[53] Yang C.-H., Wang C.-Y., Huang K.-S., Kung C.-P., Chang Y.-C., Shaw J.-F. (2014). Microfluidic one-step synthesis of Fe_3_O_4_-chitosan composite particles and their applications. Int. J. Pharm..

[54] Jangra A., Singh J., Kumar J., Rani K., Kumar P., Kumar S., Singh D., Kumar R. (2023). Dye Elimination by Surface-Functionalized Magnetite Nanoparticles: Kinetic and Isotherm Studies. Biointerface Res. Appl. Chem..

[55] Alibeigi S., Vaezi M.R. (2008). Phase Transformation of Iron Oxide Nanoparticles by Varying the Molar Ratio of Fe^2+^:Fe^3+^. Chem. Eng. Technol..

[56] Buteică S.A., Mihaiescu D.E., Pirici D., Mindrila I. (2015). Oral Fe_3_O_4_/salicylic acid nanoparticles: A rational option to the parenteral delivery. Lett. Appl. NanoBioSci..

[57] Mihaiescu D.E., Buteică A.S., Neamţu J., Istrati D., Mîndrilă I. (2013). Fe_3_O_4_/Salicylic acid nanoparticles behavior on chick CAM vasculature. J. Nanopart. Res..

[58] Holban A.M., Grumezescu A.M., Saviuc C.M., Thakur V.K., Thakur M.K. (2015). Magnetite Nanocomposites Thin Coatings Prepared by MAPLE to Prevent Microbial Colonization of Medical Surfaces. Eco-Friendly Polymer Nanocomposites: Chemistry and Applications.

[59] Ravariu C., Mihaiescu D., Morosan A., Vasile B.S., Purcareanu B. (2020). Sulpho-Salicylic Acid Grafted to Ferrite Nanoparticles for n-Type Organic Semiconductors. Nanomaterials.

[60] Ganapathe L.S., Mohamed M.A., Mohamad Yunus R., Berhanuddin D.D. (2020). Magnetite (Fe_3_O_4_) Nanoparticles in Biomedical Application: From Synthesis to Surface Functionalisation. Magnetochemistry.

[61] Banerjee R., Katsenovich Y., Lagos L., McIintosh M., Zhang X., Li C.Z. (2010). Nanomedicine: Magnetic nanoparticles and their biomedical applications. Curr. Med. Chem..

[62] Monteserín M., Larumbe S., Martínez A.V., Burgui S., Francisco Martín L. (2021). Recent advances in the development of magnetic nanoparticles for biomedical applications. J. Nanosci. Nanotechnol..

[63] Mîndrilă B., Buteică S.-A., Mîndrilă I., Mihaiescu D.-E., Mănescu M.-D., Rogoveanu I. (2022). Administration Routes as Modulators of the Intrahepatic Distribution and Anti-Anemic Activity of Salicylic Acid/Fe_3_O_4_ Nanoparticles. Biomedicines.

[64] Predoi M.C., Mîndrilă I., Buteică S.A., Purcaru Ș.O., Mihaiescu D.E., Mărginean O.M. (2020). Iron Oxide/Salicylic Acid Nanoparticles as Potential Therapy for B16F10 Melanoma Transplanted on the Chick Chorioallantoic Membrane. Processes.

[65] Mîndrilă I., Osman A., Mîndrilă B., Predoi M.C., Mihaiescu D.E., Buteică S.A. (2021). Phenotypic Switching of B16F10 Melanoma Cells as a Stress Adaptation Response to Fe_3_O_4_/Salicylic Acid Nanoparticle Therapy. Pharmaceuticals.

[66] Mîndrilă I., Buteică S.A., Mihaiescu D.E., Badea G., Fudulu A., Mărgăritescu D.N. (2016). Fe_3_O_4_/salicylic acid nanoparticles versatility in magnetic mediated vascular nanoblockage. J. Nanopart. Res..

[67] Liang X.X., Omer A.M., Hu Z.-H., Wang Y.G., Yu D., Ouyang X.-K. (2019). Efficient adsorption of diclofenac sodium from aqueous solutions using magnetic amine-functionalized chitosan. Chemosphere.

[68] Zhou Q., Wang Y., Xiao J., Fan H., Chen C. (2019). Preparation and characterization of magnetic nanomaterial and its application for removal of polycyclic aromatic hydrocarbons. J. Hazard. Mater..

[69] Nicola R., Costişor O., Ciopec M., Negrea A., Lazău R., Ianăşi C., Picioruş E.-M., Len A., Almásy L., Szerb E.I. (2020). Silica-Coated Magnetic Nanocomposites for Pb^2+^ Removal from Aqueous Solution. Appl. Sci..

[70] Abdolmohammad-Zadeh H., Salimi A. (2021). A magnetic adsorbent based on salicylic acid-immobilized magnetite nano-particles for pre-concentration of Cd(II) ions. Front. Chem. Sci. Eng..

